# Draft Genome Sequence of the Toxic Freshwater Microcystis aeruginosa Strain PMC 728.11 (Cyanobacteria, Chroococcales)

**DOI:** 10.1128/MRA.01096-20

**Published:** 2020-11-25

**Authors:** Sébastien Halary, Charlotte Duval, Alison Gallet, Sébastien Duperron, Bérénice Piquet, Justine Demay, Cécile Bernard, Benjamin Marie

**Affiliations:** a UMR 7245 MCAM MNHN-CNRS, Muséum National d’Histoire Naturelle, Paris, France; DOE Joint Genome Institute

## Abstract

Microcystis aeruginosa is one of the major species that cause toxic cyanobacterial blooms in freshwater systems worldwide. Here, we report the draft genome sequence of *M. aeruginosa* PMC 728.11, a microcystin-producing cyanobacterium isolated from the freshwater reservoir of Juanon in Valence, France. The genome sequence contains 276 contigs, consisting of 5,536,025 bp and 5,594 putative protein-coding genes, among which are several biosynthetic gene clusters encoding enzyme complexes involved in the production of various bioactive and toxic metabolites.

## Abstract (precis)

Microcystis aeruginosa is one of the major species that causes toxic cyanobacterial blooms in freshwater systems worldwide. Here, we report the draft genome sequence of *M. aeruginosa* PMC 728.11, a microcystin-producing cyanobacteria isolated from the freshwater reservoir of Juanon in Valence, France.

## ANNOUNCEMENT

*Microcystis* is among the most widespread cyanobacterial genera worldwide and is frequently reported as responsible for bloom events in freshwater environments. These blooms generally occur when water temperatures exceed 15°C in water bodies enriched by anthropogenic nutrient loading (1). Because members of this genus are able to produce diverse toxic compounds, including potent hepatotoxic microcystins, *Microcystis* recurrent blooms pose a risk for populations using impaired water resources for drinking water supplies, recreational activities, and fisheries (2). Thus, *Microcystis* strains have become good models for the investigation of ecotoxicological impacts induced by *Microcystis* blooms on aquatic organisms (3, 4).

Microcystis aeruginosa strain PMC 728.11 was isolated in September 2011 from the Juanon artificial pond (44°82′99″N, 5°01′55″E; Valence, France) during an intense bloom event. Briefly, water sample was spread onto BG11 agar plates (12:12 h light/dark cycle, 20°C), and then individual colonies were picked and grown in liquid BG11 medium. The production of microcystins was detected by enzyme-linked immunosorbent assay (ELISA) (with AD4G2 antibody; Abraxis, USA) and high-resolution mass spectrometry, together with the detection of two PCR amplicons that are commonly used as markers of its biosynthesis and corresponding to *mcyA* and *mycE* genes (5). The clonal, but nonaxenic, strain was cultured in BG-11 medium (6) at 25°C in 250-ml Erlenmeyer vessels, with a photon flux density of 12 μmol · m^−2^ · s^−1^ and a 12:12-h light/dark cycle. Total DNA extraction was carried out using a ZymoBIOMICS DNA minikit (Zymo Research, CA), and sequencing was done using 2 × 250-bp reads from both an Illumina HiSeq 2500 instrument after an initial preparation of the library (Nextera XT sample kit) and a single-molecule real-time PacBio RS II platform after library preparation with the SMRTbell library using the Express template prep kit (Pacific Biosciences). Raw reads were inspected, cut, and filtered using FastQC v0.11.5, Cutadapt v1.15, and Prinseq v0.20.4, respectively (7–9) (resulting Illumina reads: 4,948,014 reads, *N*_50_ value of 235 bp, coverage of 97×; resulting PacBio SR2 reads: 86,577 reads, *N*_50_ value of 10,986 bp, coverage of 63×). Scaffolds were assembled from HiSeq and PacBio reads using a SPAdes-based Unicycler hybrid assembler with default parameters (10, 11). Nodes from assembly graphs were clustered using MyCC (k-mer size, 4; minimal sequence size, 1,000) and taxonomically annotated using the Contig Annotation Tool (12). 16S rRNA-encoding genes were also extracted from these nodes using Metaxa 2 and then annotated using ACT (13). All contigs were pairwise aligned using MegaBLAST (E value ≤ 1e-10), and all sequences sharing a ≥98% similarity on the shortest sequence were considered as coming from the same genome. Congruent data between these diverse methodologies (binning with MyCC and BLAST with CAT) allowed us to characterize the draft genome sequence of Microcystis aeruginosa PMC 728.11.

**FIG 1:**
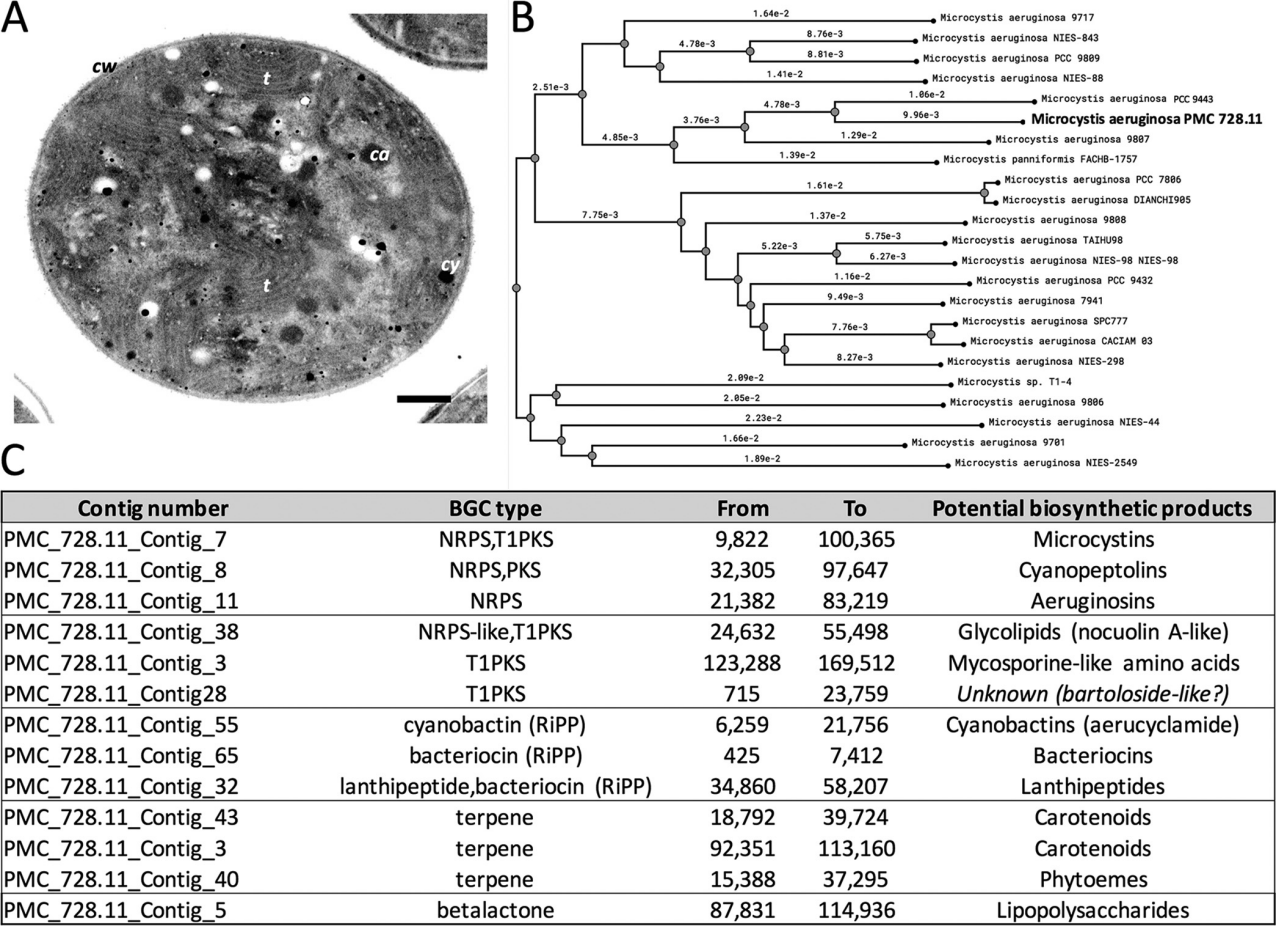
Microcystis aeruginosa strain PMC 728.11. (A) Transmission electron micrograph displaying ultrastructural details of a *Microcystis* cell, presently under division (bar, 500 nm; ca, carboxysome; cy, cyanophycine granule; cw, cell wall; t, thylakoid). (B) Phylogram of available *Microcystis* genomes based on a rapid neighbor-joining algorithm (v1.0.4) using default parameters (19) with average nucleotide identity (ANI) distances (PMC 728.11 is indicated in bold). (C) Biosynthetic gene clusters and their potential products detected by antiSMASH from a PMC 728.11 genome search, comprising gene clusters producing microcystins, among other bioactive compounds (20).

The Microcystis aeruginosa PMC 728.11 genome completeness and contamination estimated from the genomes as assessed using CheckM v1.13 with default parameters (14) were 98.57% and 1.02%, respectively. Annotation was performed using the MicroScope platform (15). The PMC 728.11 genome comprised 276 contigs (maximum length, 175,236 bp; *N*_50_, 6,601 bp; coding ratio, 78.3%) representing 5.536 Mbp, with a GC content of 42.4%. It contains potentially 5,594 gene features, including 40 tRNAs, a complete 16S-23S-5S rRNA operon, and 5 CRISPRs according to a CheckM search, these features being in perfect agreement with the 22 *Microcystis* genomes publicly available on the MicroScope server (2). Calculation of the average nucleotide identity (ANI) based on the BLAST algorithm (16) showed that PMC 728.11 displays 97.95% similarity to *M. aeruginosa* strain PCC 9443, collected in 1994 from a pond in Landjia, Central African Republic (Fig. 1B) (2).

Specialized metabolite biosynthetic gene clusters (BGCs) were identified using antiSMASH v5.1.2 (17) and MIBiG v1.4 (18). The genome harbors several gene clusters involved in the biosynthesis of various cyanopeptides, including the cyanotoxin microcystin, in addition to cyanopeptolins, aeruginosins (encoded by the nonribosomal peptide synthetase/polyketide synthase [NRPS/PKS] pathways), and cyanobactins, bacteriocins, and lanthipeptides (encoded by ribosome-synthesized posttranslationally modified peptide [RiPP] pathways), together with other genes encoding enzymes involved in the biosynthesis of glycolipids, mycosporine-like amino acids, carotenoids, phytoenes, and lipopolysaccharides (2).

In conclusion, *M. aeruginosa* strain PMC 728.11 displays features typical of toxin-producing *Microcystis*. This strain represents a promising model for further investigation and characterization of bioactive metabolites and their potential impact on organism health.

### Data availability.

The sequence of Microcystis aeruginosa PMC 728.11 has been deposited in GenBank under the BioProject accession number PRJNA650216 (GenBank accession number JADCRC010000000, BioSample number SAMN15699246, and SRA numbers SRR12746604 and SRR12746605). Strain PMC 728.11 is available from the collection of *Cyanobacteria* and Microalgae (PMC-ALCP) located in the Muséum National d’Histoire Naturelle (Paris, France; https://mcam.mnhn.fr/fr/collection-de-cyanobacteries-et-microalgues-vivantes-pmc-alcp-470).
